# Supplementary material for: Estimating the epidemic risk using non-uniformly sampled contact data
Source: Sci Rep. 2017 Aug 30;7:9975. doi: 10.1038/s41598-017-10340-y (PMC5577035; doi:10.1038/s41598-017-10340-y)
Supplement: Supplementary file 1 — Supplementary figures [file 41598_2017_10340_MOESM1_ESM.pdf]

Estimating the epidemic risk using non-uniformly sampled contact data: Supplementary Information

Julie Fournet[1], Alain Barrat[1,2,*]
[1] *Aix Marseille Univ, Université de Toulon, CNRS, CPT, 13288 Marseille, France*
[2] *Data Science Laboratory, ISI Foundation, Torino, Italy*
* *E-mail: alain.barrat@cpt.univ-mrs.fr*

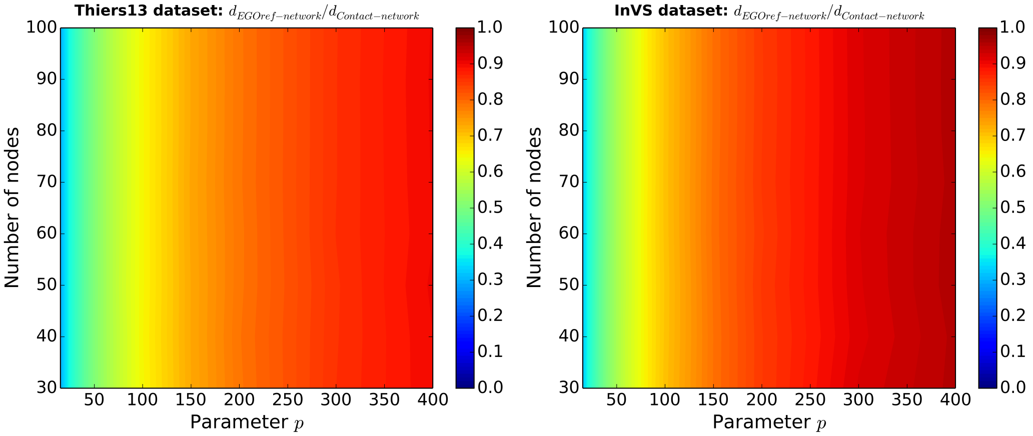

Fig. S1: Ratio between the density of the EGOref sampled network and the density of the whole contact network as a function of the parameter p and of the percentage of sampled nodes for the Thiers13 dataset (left) and the InVS dataset (right).

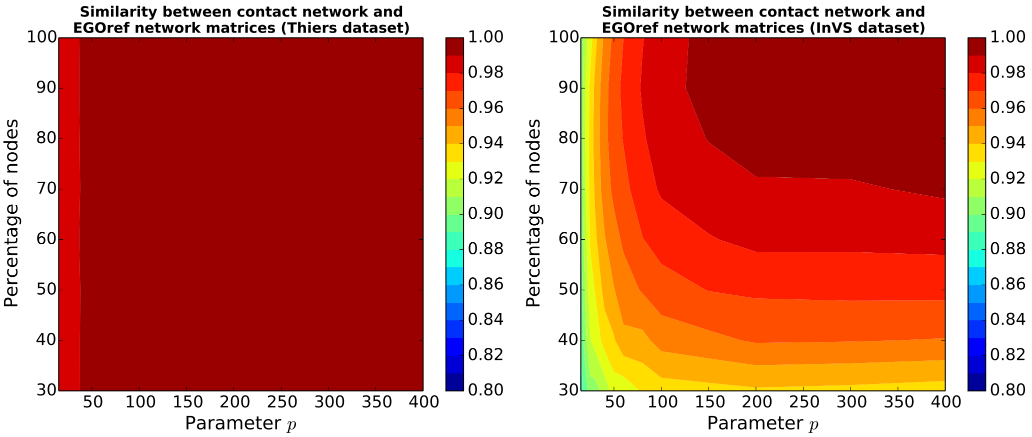

Fig. S2: Similarity between the contact matrices of the sampled and original networks, as a function of the parameter p and of the percentage of sampled nodes for the Thiers13 dataset (left) and the InVS dataset (right).

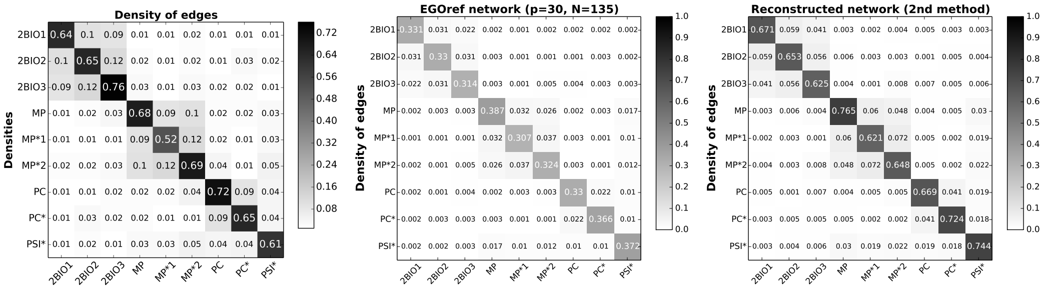

Fig. S3: Thiers13 dataset: Contact matrices giving the density of edges between departments for the contact network, the EGOref network (with $p = 30$ and N=40% of the total number of nodes) and the reconstructed network using the second method of reconstruction. The similarities between the three matrices are all above 98%.

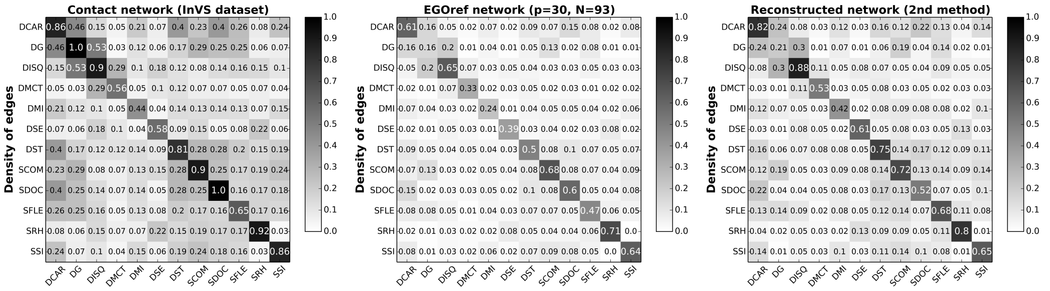

Fig. S4: InVS dataset: Contact matrices giving the density of edges between departments for the contact network, the EGOref network (with $p = 30$ and N=40% of the total number of nodes) and the reconstructed network using the second method of reconstruction. Similarity between the matrix of the contact network and of the EGOref network: 93%, between the matrix of the contact network and of the reconstructed network: 98%, between the matrix of the EGOref network and of the reconstructed network: 94%.

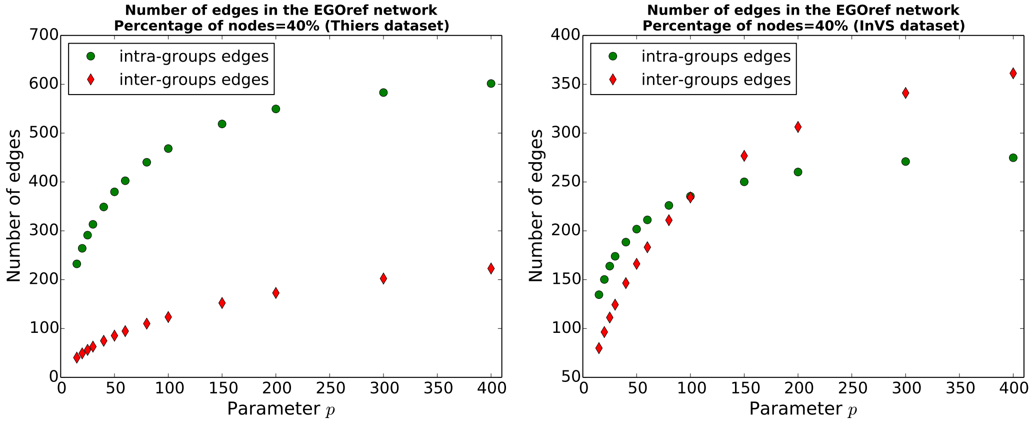

Fig. S5: Number of intra-class and inter-class edges for Thiers13 and InVS in the sampled network, at varying p and for N = 40%.

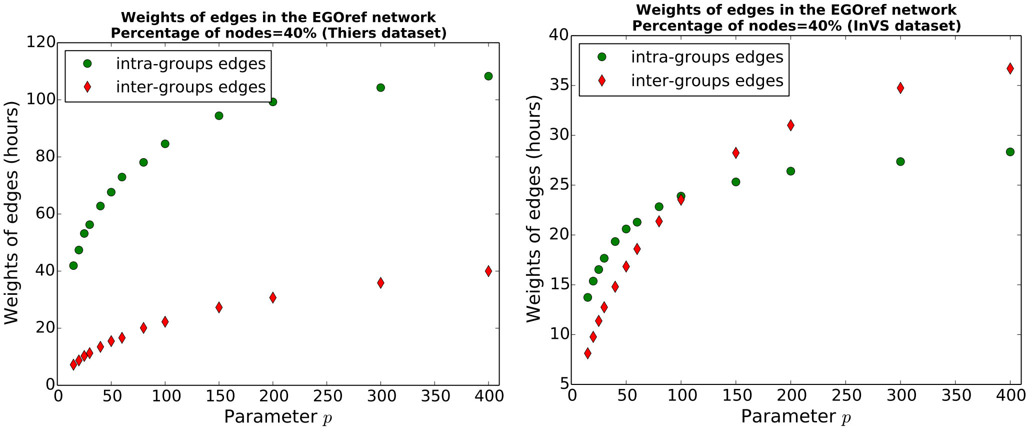

Fig. S6: Total weight carried by intra-class and inter-class edges for Thiers13 and InVS in the sampled network, at varying p and for N = 40%.

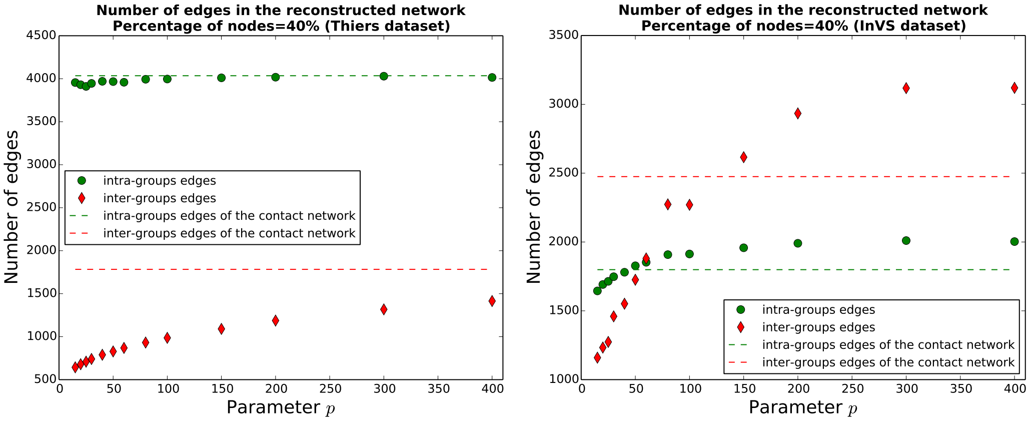

Fig. S7: Number of intra-class and inter-class edges for Thiers13 and InVS in the reconstructed network, at varying p and for N = 40%. The horizontal lines give the values in the original data.

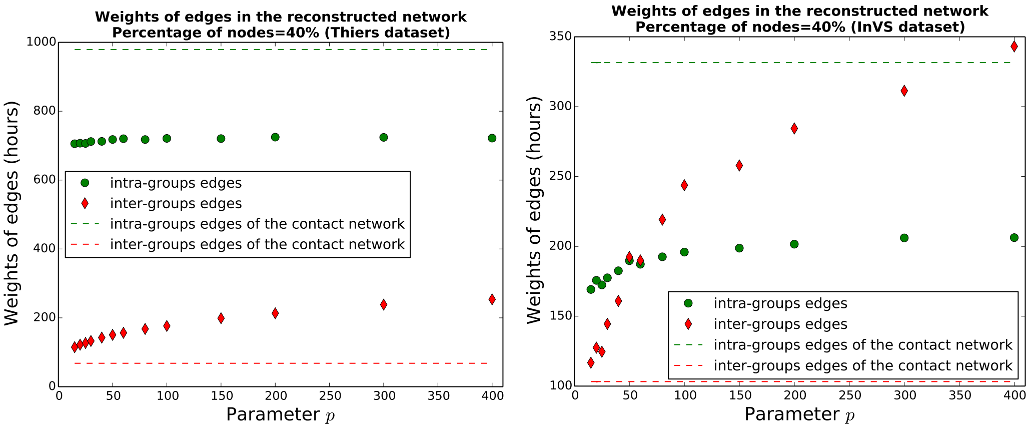

Fig. S8: Total weight carried by intra-class and inter-class edges for Thiers13 and InVS in the reconstructed network, at varying p and for N = 40%. The horizontal lines give the values in the original data.

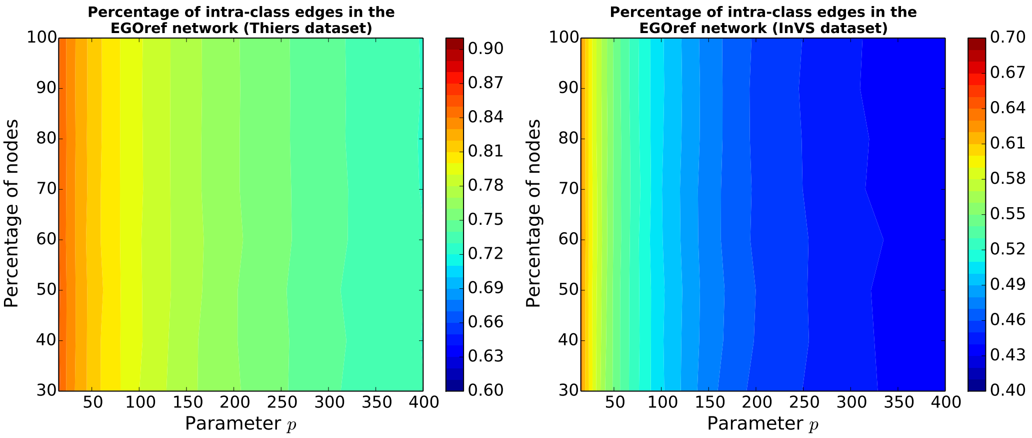

Fig. S9: Fraction of intra-class edges as a function of the sampling parameter p and of the percentage of sampled nodes for the Thiers13 dataset (left) and the InVS dataset (right).

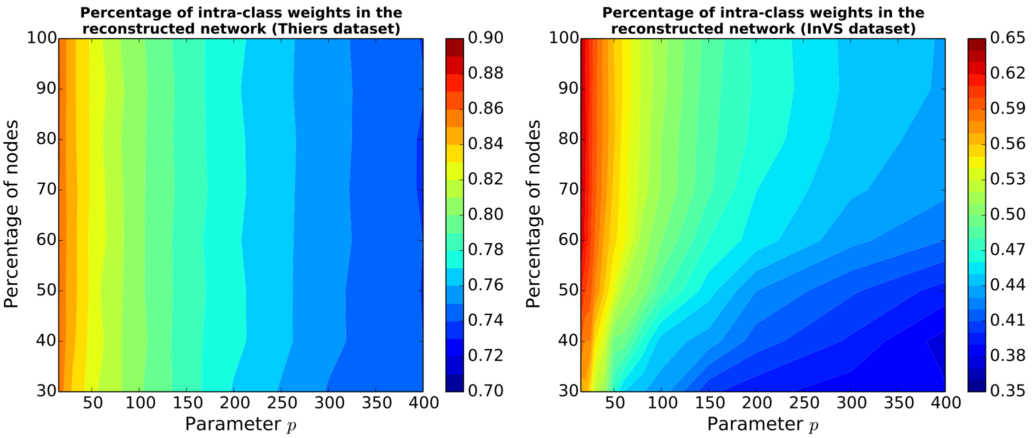

Fig. S10: Fraction of the total weight carried by intra-class edges in the surrogate data as a function of the sampling parameter p and of the percentage of sampled nodes for the Thiers13 dataset (left) and the InVS dataset (right).
